# Exploring the secrets of virus entry: the first respiratory syncytial virus carrying beta lactamase

**DOI:** 10.3389/fmicb.2024.1339569

**Published:** 2024-02-16

**Authors:** Marcio De Ávila-Arias, Jose Luis Villarreal-Camacho, Christian Cadena-Cruz, Leidy Hurtado-Gómez, Heather M. Costello, Alexander Rodriguez, Francisco Burgos-Florez, Alfonso Bettin, Meisam Naeimi Kararoudi, Amner Muñoz, Mark E. Peeples, Homero San-Juan-Vergara

**Affiliations:** ^1^Departamento de Medicina, División Ciencias de la Salud, Universidad del Norte, Barranquilla, Colombia; ^2^Programa de Medicina, Facultad de Ciencias de la Salud, Universidad Libre Seccional Barranquilla, Barranquilla, Colombia; ^3^Genomics Services Laboratory, Abigail Wexner Research Institute at Nationwide Children’s Hospital, Columbus, OH, United States; ^4^Programa de regencia en farmacia, grupo de investigación creatividad e innovación tecnológica, Corporación tecnológica Indoamérica, Barranquilla, Colombia; ^5^Escuela de Pregrado, Dirección Académica, Vicerrectoría de Sede, Universidad Nacional de Colombia, Sede La Paz, Cesar, Colombia; ^6^Center for Childhood Cancer and Blood Diseases, Abigail Wexner Research Institute at Nationwide Children’s Hospital, Columbus, OH, United States; ^7^Departamento de Química y Biología, Universidad del Norte, Barranquilla, Colombia; ^8^Center for Vaccines and Immunity, The Abagail Wexner Research Institute at Nationwide Children’s Hospital, Columbus, OH, United States

**Keywords:** respiratory syncytial virus, reporter virus, viral entry mechanisms, quercetin, ERK (extracellular signal-regulated kinase)

## Abstract

**Background:**

Respiratory Syncytial Virus (RSV) presents a significant health threat, especially to young children. In-depth understanding of RSV entry mechanisms is essential for effective antiviral development. This study introduces an innovative RSV variant, featuring the fusion of the beta-lactamase (BlaM) enzyme with the RSV-P phosphoprotein, providing a versatile tool for dissecting viral entry dynamics.

**Methods:**

Using the AlphaFold2 algorithm, we modeled the tertiary structure of the P-BlaM chimera, revealing structural similarities with both RSV-P and BlaM. Functional assessments, utilizing flow cytometry, quantified beta-lactamase activity and GFP expression in infected bronchial epithelial cells. Western blot analysis confirmed the integrity of P-BlaM within virions.

**Results:**

The modeled P-BlaM chimera exhibited structural parallels with RSV-P and BlaM. Functional assays demonstrated robust beta-lactamase activity in recombinant virions, confirming successful P-BlaM incorporation as a structural protein. Quercetin, known for its antiviral properties, impeded viral entry by affecting virion fusion. Additionally, Ulixertinib, an ERK-1/2 inhibitor, significantly curtailed viral entry, implicating ERK-1/2 pathway signaling.

**Conclusions:**

Our engineered RSV-P-BlaM chimera emerges as a valuable tool, illuminating RSV entry mechanisms. Structural and functional analyses unveil potential therapeutic targets. Quercetin and Ulixertinib, identified as distinct stage inhibitors, show promise for targeted antiviral strategies. Time-of-addition assays pinpoint quercetin’s specific interference stage, advancing our comprehension of RSV entry and guiding future antiviral developments.

## Introduction

Respiratory syncytial virus (RSV), a member of the orthopneumovirus genus, whose genome is a negative-polarity, single-stranded RNA. It infects approximately 60 million people worldwide each year, resulting in an estimated 160,000 deaths (Mohapatra and Boyapalle, 2008). Despite various initiatives offering hope for vaccine development, no vaccines have been made available for use thus far.

The viral entry mechanisms and the ribonucleoprotein release are only partially understood. The RSV envelope contains three proteins encoded in the viral genome: G (attachment), F (fusion), and SH (small hydrophobic protein) (Liljeroos et al., 2013). The G protein, a mucin-like protein, appears to interact with the fractalkine receptor (Johnson et al., 2015; Anderson et al., 2020). The F protein mediates fusion between the virus envelope and cell membranes, with nucleolin being one of the proposed cellular receptors facilitating F binding, triggering a conformational change exposing the fusion peptide (Griffiths et al., 2020). The SH protein seems unrelated to the entry process and may function as a viroporin (Gan et al., 2012).

It has been suggested that host cells also contribute to RSV entry through mechanisms that would depend on endocytosis or changes in the cell membrane (Krzyzaniak et al., 2013). However, the conclusions drawn so far have relied on approaches involving recombinant virions expressing fluorescent or luminescent proteins, making it difficult to definitively determine which of these processes are associated with entry. Additionally, directly evaluating the screening of compounds with antiviral activity targeting RSV entry or content release has been challenging.

In this study, we have developed a recombinant RSV virion carrying the enzyme beta-lactamase (BlaM) fused to the C-terminus of the RSV P phosphoprotein. The release of the ribonucleoprotein can be detected by the enzymatic activity of BlaM, which cleaves the beta-lactam ring on the CCF2 fluorescent substrate, a bridge between hydrocoumarin and fluorescein, increasing the fluorescence of the hydroxycoumarin. This strategy was designed by Cavrois’s et al. (2002) work using BlaM associated with the HIV-1 VPR protein to study HIV-1 entry mechanisms. However, the use of BlaM has so far been limited to pseudotyped virions or virus-like particles. By employing rgRSV-P-BlaM (RSV carrying BlaM), We demonstrated that quercetin inhibits virion entry similar to palivizumab, and that ERK-1/2 inhibition affects virion entry in human bronchial epithelial cells.

The incorporation of BlaM into the native RSV virion enables direct conclusions regarding the cellular components contributing to ribonucleoprotein release and facilitates the screening and identification of pharmacologically active molecules capable of blocking RSV entry. This approach may hold promise for further understanding RSV infection mechanisms and for developing potential therapeutic interventions.

## Materials and methods

### Cells

Baby Hamster Kidney cells (BHK-21, Kerafast, Cat # EH1011), Lenti-X Human Embryonic Kidney cells (Lenti-X HEK-293T, Clontech, Cat # 632180), Normal Human Bronchial Epithelial cells (NHBE, Lonza, Cat # CC-2541) and HEp-2 cells (ATCC, Cat # CCL-23) were maintained in the respective growth medium and incubated at 37°C, 5% CO_2_ atmosphere and 95% relative humidity.

BHK-T7 cells constitutively expressing T7-RNA polymerase were developed using lentiviral transduction. Cells were used for the production of the rgRSV-P-BlaM recombinant virus. BHK-21, BHK-T7 and Lenti-X HEK-293T were grown in DMEM supplemented with 10% heat-inactivated Fetal Bovine Serum (FBS) (Gibco BRL, Carlsbad, CA, United States), 100 U/mL penicillin, 100 μg/mL streptomycin. BHK-21 and BHK-T7 required an additional supplement of 2 mM L-glutamine; while the culture medium of Lenti-X HEK-293T required to be supplemented with 1 mM sodium pyruvate.

HEp-2 Cells were obtained from American Type Culture Collection (ATCC number: CCL-23) and maintained in Opti-MEM (Gibco BRL, Carlsbad, CA, United States) supplemented with 10% FBS, 100 U/mL penicillin, 100 μg/mL streptomycin.

Undifferentiated bronchial epithelial cells (NHBE) were maintained in Bronchial Epithelial Growth Medium (BEGM) (Lonza, Walkersville, MD, United States) following the supplier’s instructions.

### Plasmids

The plasmid MDA-P-BlaM containing the RSV cDNA with the coding sequence for P-BlaM was derived from plasmid HC123 (Hallak et al., 2000). The P-BlaM as an extra sequence was constructed from gBlocks (Integrated DNA Technologies). The BlaM sequence is an optimized version of beta lactamase Y105W (Wolf et al., 2009). Using Gibson Assembly (New England Biolabs), gBlocks were cloned between P gene and M gene using AvrII (nt. 2930) and PvuI (nt. 6573) restriction sites of RSV HC123 cDNA plasmid. The plasmid MDA-P-BlaM is designed with a GFP gene positioned as the initial gene preceding the NS1 gene, and the entire vector is regulated by the T7 promoter. Plasmid pLVX-Hyg-T7 resulted by inserting the coding sequence T7 RNA polymerase (gift of Dr. Guy Lemay, University of Montreal, Canada) between XhoI and NotI restriction sites of plasmid pLVX-IRES-Hyg (Clontech Inc., Mountain View, CA, United States). The pLVX-Hyg-T7 plasmid was used to generate BHK-T7 cells. pLVX-Hyg-P-BlaM was constructed by inserting the P-BlaM coding sequence using gBlocks (Integrated DNA Technologies) into XhoI and NotI restriction sites of the pLVX-IRES-Hyg construct (Clontech Inc., Mountain View, CA, United States).

Plasmids were electroporated into *Escherichia coli* EPI300 (Lucigen Corp., Middleton, WI, United States, Catalog EC300110) for amplification. Plasmid purification was performed using QIAprep Spin Miniprep (QIAGEN GmbH, Hilden, Germany) and HiPure Plasmid Maxiprep (Invitrogen, Carlsbad, CA, United States).

### Lentiviruses pVLX-T7 and pVLX-P-BlaM

Plasmids containing sequences of interest were mixed with Lenti-X HTX Packaging mix (Clontech Inc., Mountain View, CA, United States), followed by transfection into Lenti-X HEK-293T cells in the presence of Xfect (Clontech Inc., Mountain View, CA, United States), following the manufacturer’s instructions. The lentiviral particles pVLX-T7 and pVLX-P-BlaM were recovered and frozen at −150°C for later use.

### BHK-T7 and Hep-2 P-BlaM cell lines

BHK-21 cells and HEp-2 cells were transduced with respective lentiviruses to generate the BHK-T7 and Hep-2-P-BlaM cell lines, respectively. The transduction was performed using spinoculation; where, 100 μL of the lentiviral suspension was added to the cells followed by centrifugation at 1,200 g for 90 min at 32°C. The inoculum was removed. Cell culture was washed with DPBS and then maintained in DMEM supplemented with 2% FBS for 24 h. Transduced cells were selected for their resistance to hygromycin B (200 μg/mL). The selection process took 15 days, changing the medium supplemented with hygromycin every 48 h. BHK-T7 and Hep-2-P-BlaM cells were cryopreserved in DMEM medium supplemented with 10% DMSO and 10% FBS.

### RSV P-BlaM (rgRSV-P-BlaM) rescue and production

BHK-T7 cells were transfected with the plasmid MDA-P-BlaM and helper plasmids pA2-Nopt, pA2-M2-1opt, pA2-Lopt, pA2-Popt (Hotard et al., 2012), using Lipofectamine 3,000 (Thermo Fisher Scientific). After 48 h, the transfected cells were subcultured in a 25 cm^2^ flask (Corning^®^). Once the syncytia reached out 60% of the monolayer, the cells were separated using a scraper in a volume of 5 mL of culture medium. Cell debris was removed by centrifugation at 1,800 g for 10 min at 4°C. The virions present in the supernatant were stabilized by the dropwise of MgSO_4_ (0.1 M). Virions were cryopreserved at −150°C.

The aliquots containing recovered virions were used to propagate the rgRSV-P-BlaM virions according to Hallak et al. (2000). One 1 milliliter aliquot of rgRSV-P-BlaM was used to infect HEp-2 cells grown in a 75 cm^2^ flask. After 2 h of incubation at 37°C, the medium was replaced by DMEM supplemented with 2% FBS and 1% Penicillin-Streptomycin, with medium changes every 48 h until fluorescent syncytia were observed that reached 60% of the monolayer. The supernatant containing the recombinant virus was cryopreserved at −150°C.

### Evaluation of the ability of RSV to carry P-BlaM produced in trans

Hep-2-P-BlaM cells were infected following the previously described methodology. Viral titer by GFP and P-BlaM activity were characterized by flow cytometry and western blot, respectively.

### Viral titer quantification

The viral titer was determined following the protocol of Techaarpornkul et al. (2001). Briefly, NHBE cells grown in 24-well dishes were infected with serial dilutions of aliquots containing the infectious inoculum for 2 h at 37°C. then, the inoculum was removed and cells were incubated for 16 h at 37°C. Infected cells were detected by expression of the GFP signal; while the cells where there was release of the ribonucleoprotein content of the virus was estimated by the signal of the fluorescence shift towards the hydroxycoumarin channel by indicating the beta-lactam ring cleavage into CCF2 fluorochrome. The viral titer was estimated considering that dilution of the aliquot that generated a signal in the range of 0.05 to 0.1 of the total cells that were analyzed by flow cytometry.

### Presence of the P-BlaM protein in virions by western-blot

The rgRSV-P-BlaM viral suspension was centrifuged at 20,000 g for 2 h at 4°C to concentrate the virions in a smaller volume. The proteins present in the virions were obtained by lysis. Used as a control, lysate from a culture of cells infected with rgRSV-P-BlaM was used as a positive control. Nupage LDS sample buffer (Thermo Fisher) was used for the lysate in the presence of Halt^™^ Protease Inhibitor Cocktail (Thermo Fisher). Proteins were separated by SDS-PAGE under reducing conditions (+βME) and transferred to a nitrocellulose membrane. The P-BlaM protein was detected with the Anti-BlaM antibody [clone 8A5.A10] (ab12251). The binding of anti-BlaM was revealed by secondary antibodies labeled with near-infrared fluorochromes (800cw goat anti mouse IgG1) and visualized with Odyssey (LI-COR, Lincoln, NE, United States).

### Modeling of P-BlaM chimera

The P-BlaM protein was modeled *in silico* using the Colab server (https://colab.research.google.com/github/sokrypton/ColabFold/blob/main/AlphaFold2.ipynbaccessed 01/06/2022). The generated structure was compared to protein P (Uniprot code P03421) and BlaM (Uniprot code C5I4X2). The reliability of the system predictions was assessed by the Local Distance Difference Test (LDDT) score according to the CASP14 assessment steps published by Jumper et al. (2021). The intrinsically disordered regions identified by AlphaFold2 were reviewed using DISOPRED3 (PSIPRED Workbench ucl.ac.uk) (Jones and Cozzetto, 2015). In addition, the amino acid sequence of the P protein was also modeled in AlphaFold2 and compared with the modeled structure of the P-BlaM chimera. The structure of the P protein was also reviewed using the derived model from cryo-EM studies that analyzed the P-protein tetramer in interaction with the RSV-L protein (PDB: 6PZK) (Gundenon et al., 1987).

### Evaluation of quercetin effect on viral entry using time-of-addition assays

NHBE cell cultures were exposed to an infectious inoculum of rgRSV-P-BlaM at an infectious dose of 3 FFU per 10 cells, which is approximately 0.3 MOI, for 1 h at 22°C This temperature allowed the adsorption but not the fusion (Henderson and Hope, 2006). The fusion was triggered by switching the temperature to 37°C, which consequently was defined as time “0.” Just before switching the temperature, we replaced medium in all cultures. For the set labeled as time “0.” A set of 3 dishes were incubated with quercetin (125 μg/mL) or with palivizumab (200 μg/mL) or mock. At each intervention time, the medium in the respective set of cultures was replaced with the one with the experimental condition. This was done until we reached 120 min. After 120 min, all the dishes were incubated with HBSS supplemented with 2 μM CCF2-AM (GeneBLAzer *in vivo* Detection Kit, Thermo Fisher Scientific) and the culture was incubated for an additional 1 h at 17°C. The cells were processed by flow cytometry (BD FACSCanto II) and hydroxycoumarin signal was measured for each condition.

### Evaluation of ulixertinib effect, an ERK-1/2 inhibitor, on viral entry

NHBE cells grown in 24-well dishes to 80% confluence, were treated with different concentrations of ulixertinib for 1 h at 37°C. Cultures were inoculated with rgRSV-P-BlaM at an equivalent dose of 1 virion per 2 cells, in the presence of the inhibitor. The virions were adsorbed on the plasma membrane for 1 h at 22°C, to synchronize the viral entry at 37°C. After 2 h, all wells were incubated with HBSS supplemented with 2 μM CCF2-AM (GeneBLAzer *in vivo* Detection Kit, Thermo Fisher Scientific), for 1 h at 17°C. The cells were processed by flow cytometry and hydroxycoumarin signal was measured for each condition.

## Results

### Modeling of the P-BlaM chimera

**Figure 1:**
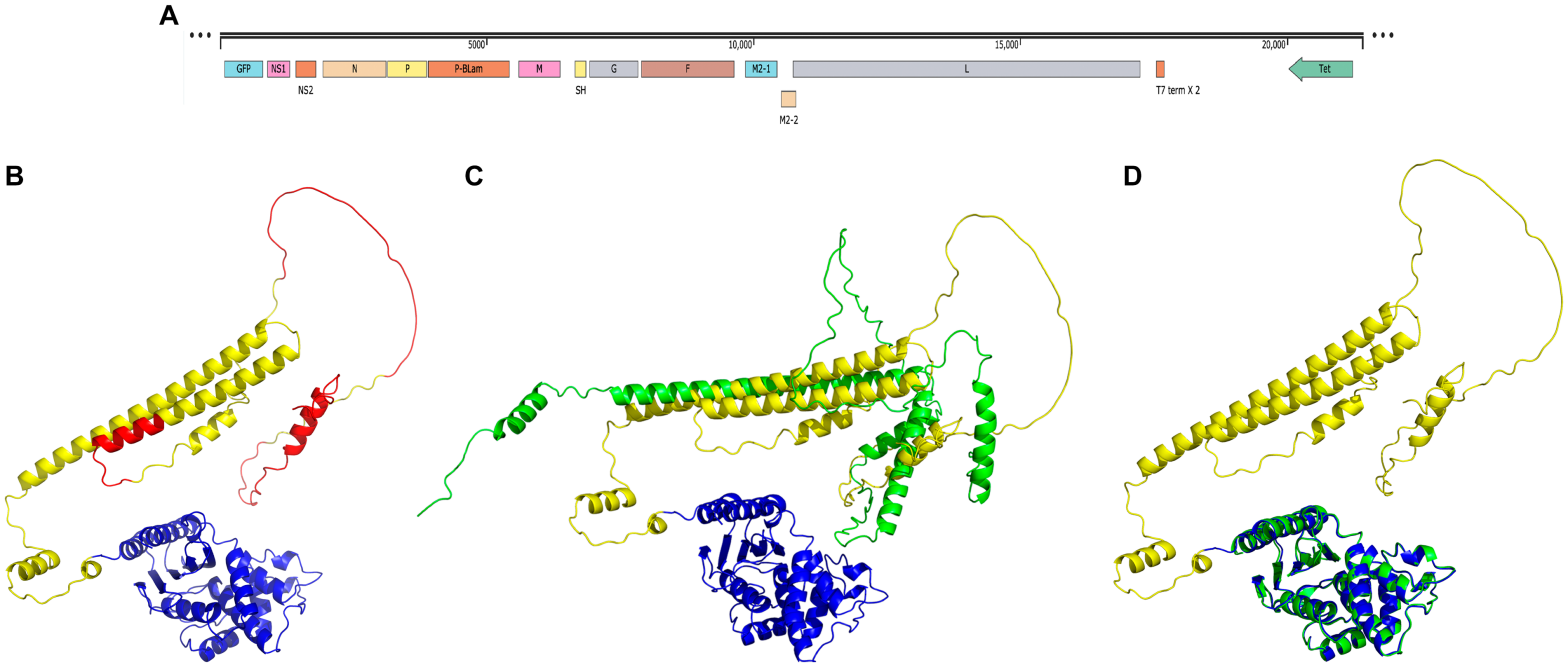
Structural homology of the P-BlaM chimera with the respective RSV-P and BlaM proteins. **(A)** Scheme of the modified cDNA of the rgRSV-P-BlaM genome. The sequence encoding P-BlaM is located as an extra gene immediately downstream of the gene encoding RSV-P. GFP gene is positioned as the initial gene preceding the NS1 gene. The cDNA construct is regulated by the T7 promoter. **(B)** Modeled structure of the P-BlaM chimera protein using AlphaFold2. The BlaM corresponding domain is colored blue. The RSV-P domain is labeled with yellow-green and red colors. The red color indicates intrinsically disordered regions according to the Disopred3 software. **(C)** Comparison of the modeled structures of the P-BlaM chimera with the RSV-P protein. **(D)** Spatial alignment of the crystal structure of the BlaM protein (PDB: 1XPB) elucidated by X-ray diffraction in blue with the BlaM protein from the chimera modeled with AlphaFold in green.

The MDA-P-BlaM plasmid containing the RSV cDNA was modified by inserting the recombinant P-BlaM sequence between the P and M genes (Figure 1A). The tertiary structure of the P-BlaM chimera was modeled using the Colab server running the AlphaFold2 algorithm (https://colab.research.google.com/github/sokrypton/Colab/blob/main/AlphaFold2.ipynb accessed 01/06/2022) (Figure 1B). The modeled structure was visualized in PyMOL. The modeled structure of the chimera RSV-P phosphoprotein has a 5 α helices and 5 loops in common as modeled sequence from the RSV-P phosphoprotein. Regions that share a loop-like structure include: 1M-10G, T29-N92, N111-N124, D212-N217, and S232-S237; while those that has similar α-helices are: E11-I24, S99-F109, I131-A155, T160-K208 and P218-L227 (Figure 1C). Comparing the modeled structure of the chimera with the regions to which we were able to access one of the monomers reported by Gilman et al. (2019), and which is deposited in the protein databank (PDB: 6PZK), we found homology in the respective alpha-helice zones; among these: 131ITARLDRIDEKLSEILGMLH151, 174REEMIEKIRT183, 190DRLEAMARLR199, 203SEKMAKDT210 and 217NPTSEKLNNLLE228. Likewise, the loop region 211SDEVSL216 is also shared. The regions that show disparity when both structures are compared as 161SARDGIRDAMVGL173 and 184EALMTN189. In the chimera P protein, these regions adopt an α-helical structure, while in the P protein elucidated by cryo-MS they have a loop shape.

In a study performed by Gilman et al. (2019), it is observed that phosphoprotein P shows structural plasticity of such magnitude that it has been proposed as an intrinsically disordered protein. The P protein has a low representation of hydrophobic amino acids (19.9%) and aromatic amino acids (5%), which is compatible with what is expected for intrinsically disordered proteins. In addition, Gly and Pro constitute 8.3% of the amino acids of the RSV-P protein, which are characterized by destroying the α-helices and β-sheet secondary structures. The presence of regions without a defined secondary structure in the RSV-P protein of the chimera is compatible with the characteristics of an intrinsically disordered protein (Knauer et al., 2012), like what was previously described for the RSV NS1 and NS2 proteins.

The structure obtained by crystallography was retrieved from the Protein Databank using the keyword beta-lactamase and aligned with the structure of the P-BlaM chimera protein modeled by AlfaFold2 in PyMOL. The structure of the chimera BlaM has a high structural homology with respect to the reported structure PDB: 1XPB (Figure 1B vs. Figure 1D), where the BlaM chimera protein retains all 12 α helices, 7 folded β sheets and 5 turns. This indicates that the P-BlaM chimera protein retains the conformational structure compatible with the preservation of BlaM enzymatic activity.

### Functional evaluation of the beta-lactamase activity of recombinant viruses rgRSV-P-BlaM

Undifferentiated bronchial epithelial cell cultures were inoculated with recombinant rgRSV-P-BlaM viruses for 1 h at 22°C to synchronize the entry of the virions. Once the infection was carried out at 37°C for 2 h, the culture medium was replaced with a loading buffer containing the CCF2-AM substrate. The CCF2-AM loading was performed at 17°C for 3 h, after which the cells were analyzed using flow cytometry to evaluate the beta-lactamase activity of the recombinant viruses.

**Figure 2:**
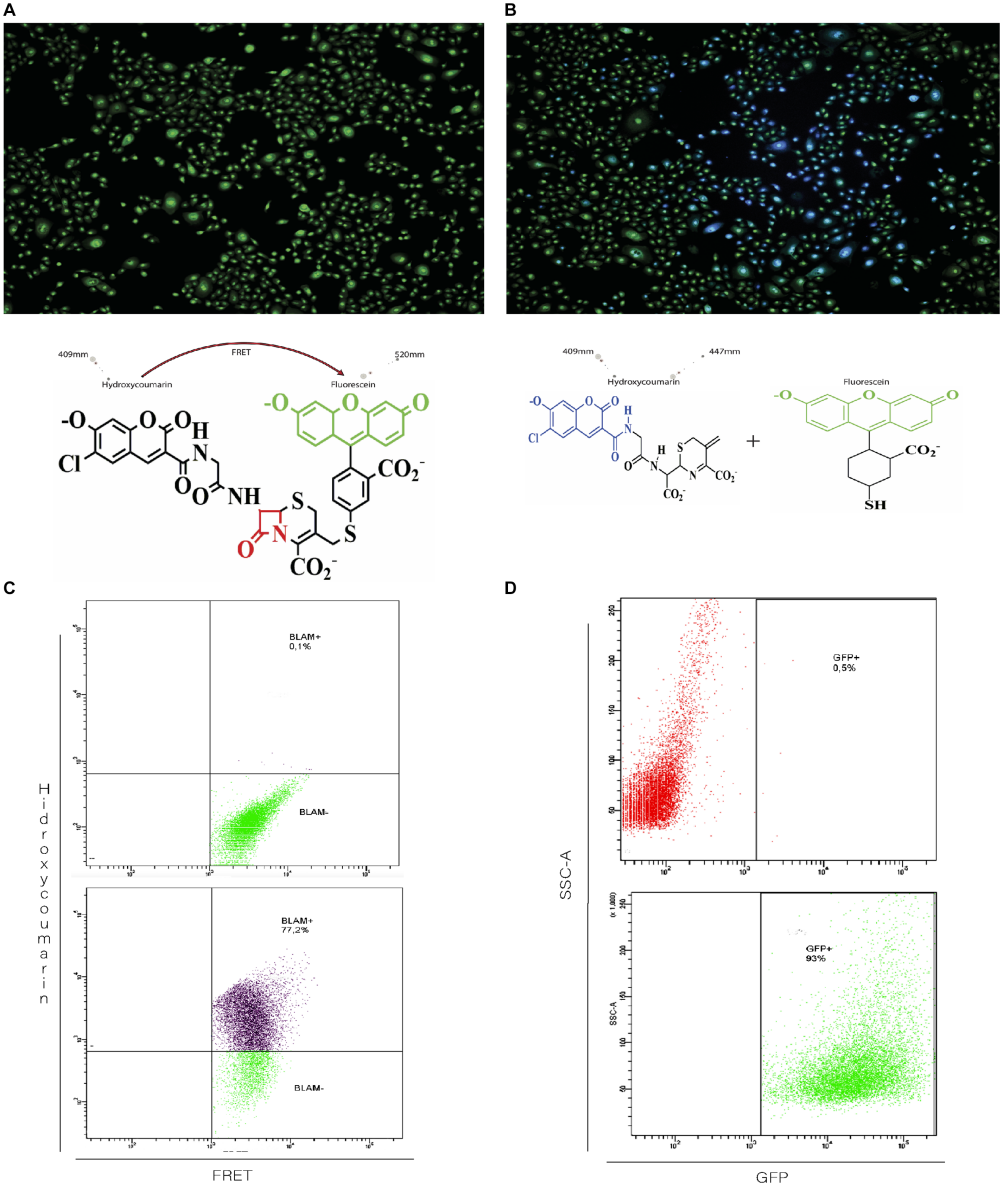
Functional evaluation of beta-lactamase activity in recombinant rgRSV-P-BlaM virions. **(A,B)** Beta-lactamase activity evidenced by the shift in fluorescence emission corresponding to hydroxycoumarin. NHBE cultures were loaded with CCF2 in the absence (**A**, left image) or after incubation with the infectious rgRSV-P-BlaM inoculum (**B**, right image) for 2 h. In the absence of the infectious inoculum **(A)**, hydroxycoumarin excitation generates emission in the fluorescein channel (cells in green) due to FRET. Due to the presence of beta-lactamase in infected cells **(B)**, the beta-lactam ring is cleaved, and the emission occurs in the hydroxycoumarin channel (cells in blue). **(C,D)** Flow cytometry analysis of beta-lactamase activity and reporter GFP expression. In **(C)**, the percentage of NHBE cells that shifted to the hydroxycoumarin emission channel as a result of infection for 2 h is shown. In **(D)**, the percentage of NHBE cells expressing GFP from an equivalent volume aliquot used in **(C)** is shown.

The CCF2-AM fluorescent dye is a molecule composed of hydroxycoumarin and fluorescein linked with a beta-lactam ring acts a bridge. In the absence of beta-lactamase, the excitation of hydroxycoumarin at 409 nm generates emission associated with fluorescein (520 nm) due to the Föster emission resonance transfer (FRET) phenomenon. In Figure 2A, it is observed that in uninfected cultures, all cells show green fluorescence resulting from the emission of fluorescein.

On the other hand, infected cells from cultures exposed to rgRSV-P-BlaM show blue fluorescence because the presence of beta-lactamase cleaves the beta-lactam ring, resulting in the disruption of FRET between hydroxycoumarin and fluorescein. Thus, when stimulating infected cells at 409 nm, they emit fluorescence at 447 nm (Figure 2B).

Considering that the rgRSV-P-BlaM virion also encodes the GFP fluorescent protein, we evaluated whether the percentage of cells showing beta-lactamase activity correlated with the percentage of cells expressing GFP. The shift in fluorescence signal to hydroxycoumarin was identified in cells derived from cultures exposed to the infectious aliquot for 2 h, using flow cytometry. At the same time, other cultures were infected with the same volume of aliquot, and the percentage of cells expressing EGFP was evaluated 16 h later. The percentages obtained through both approaches were identical (Figure 2C vs. Figure 2D).

Regarding the substantial diversity of GFP-expressing cells, we must emphasize the following: (1) although the RSV genome contains the genes encoding GFP and P-BlaM, the experimental design assessed different things. Regarding BlaM, we detected its activity in the cells after its delivery during the entry phase by virions carrying it as a structural protein. On the other hand, GFP was assessed 16 h later in a concurrent and parallel set of cultures using the same aliquot and infectious dose; consequently, we detected GFP expression. (2) RSV is pleomorphic and both filamentous and spherical particles have been reported. Structural analysis of RSV using cryo-electron tomography revealed that the number of genome copies varies significantly among viral particles in the same aliquot (Kiss et al., 2014). In spherical viral particles, Kiss et al. (2014) reported up to 9 genome copies per particle, while the filamentous particles were reported to carry up to 3 copies. Thus, the substantial diversity in GFP fluorescence intensity is the expected result of cells being infected by virions carrying different numbers of genome copies. In addition, viral infection follows a Poisson distribution; that is, cells are not equally infected by the same number of virions (Shabram and Aguilar-Cordova, 2000).

### The P-BlaM protein is a structural protein in the virion

**Figure 3:**
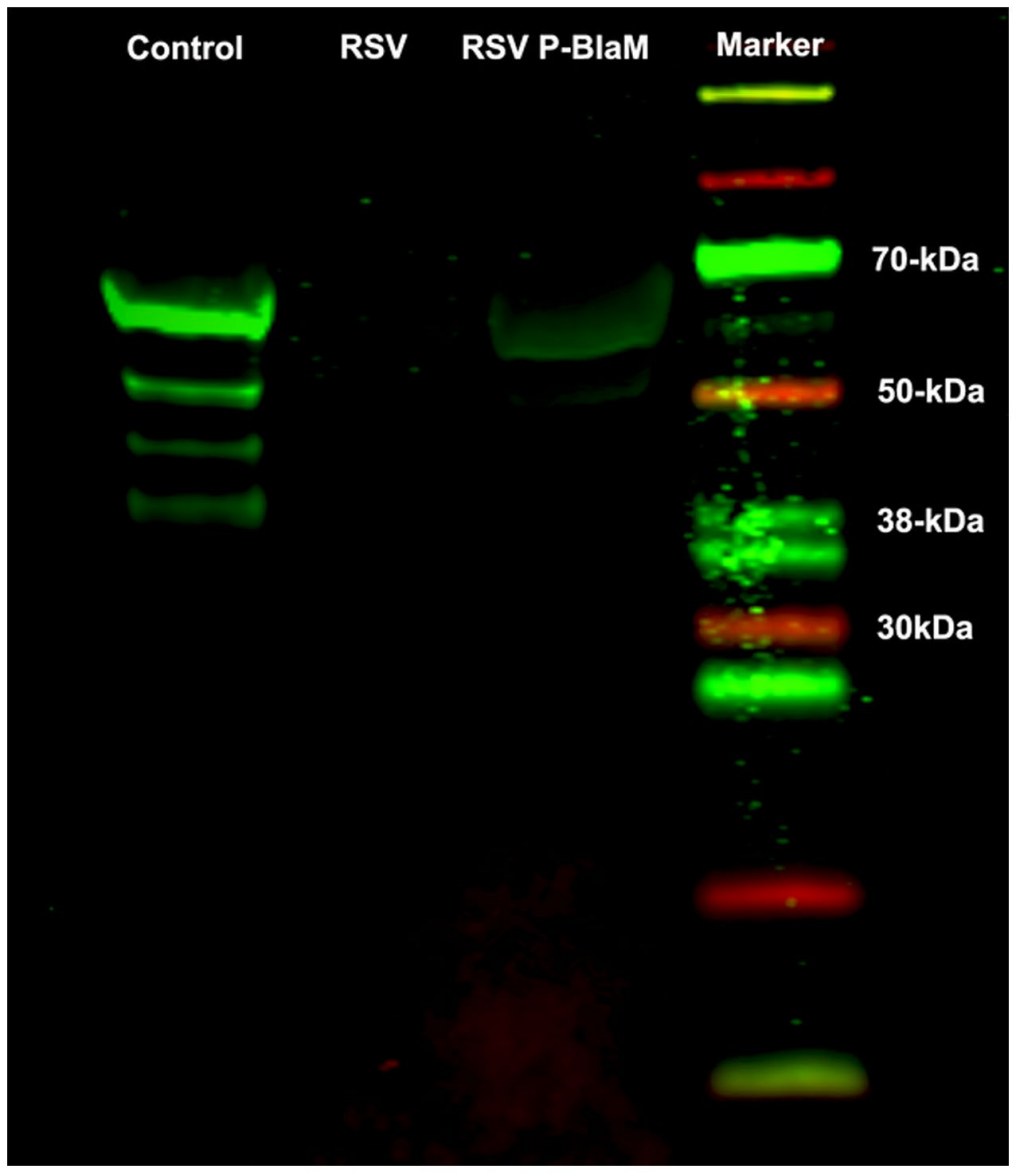
Presence of the P-BlaM protein in the preparation of recombinant virions. Lysates of the following preparations were run on SDS-PAGE: NHBE cells infected with rgRSV-P-BlaM, suspension of rgRSV virions, and suspension of rgRSV-P-BlaM virions. After respective transfer to a nitrocellulose membrane, the presence of P-BlaM in the respective lanes was identified using a primary monoclonal antibody against BlaM. Using the LiCor system, we used 800cw goat anti-mouse IgG1 as secondary antibodies and visualized in the infrared end on Odyssey. A set of differently molecular-weighted proteins labeled to be visualized in the infrared end was also run.

The presence of the P-BlaM protein in the virions was detected using an anti-BlaM antibody labeled with a near-infrared fluorescence tag in a western blot system (Figure 3). Routledge et al. (1988) found that the RSV-P phosphoprotein ran on a gel as a ladder of polypeptides with different molecular weights (19 kDa to 34 kDa), with the most abundant band corresponding to a molecular weight of 34 kDa. The apparent molecular weight for BlaM is 27.2 kDa. Consequently, the apparent molecular weights of the bands observed in the control lane, where the lysate of the rgRSV-P-BlaM-infected culture ran, matched the expected range for the P-BlaM polypeptide ladder (46.2 kDa to 61.2 kDa). In the lane where the lysate of virions isolated from rgRSV-P-BlaM ran, a single band at the level of 61.2 kDa was observed, which was not present in the lane with RSV virions’ lysate.

**Figure 4:**
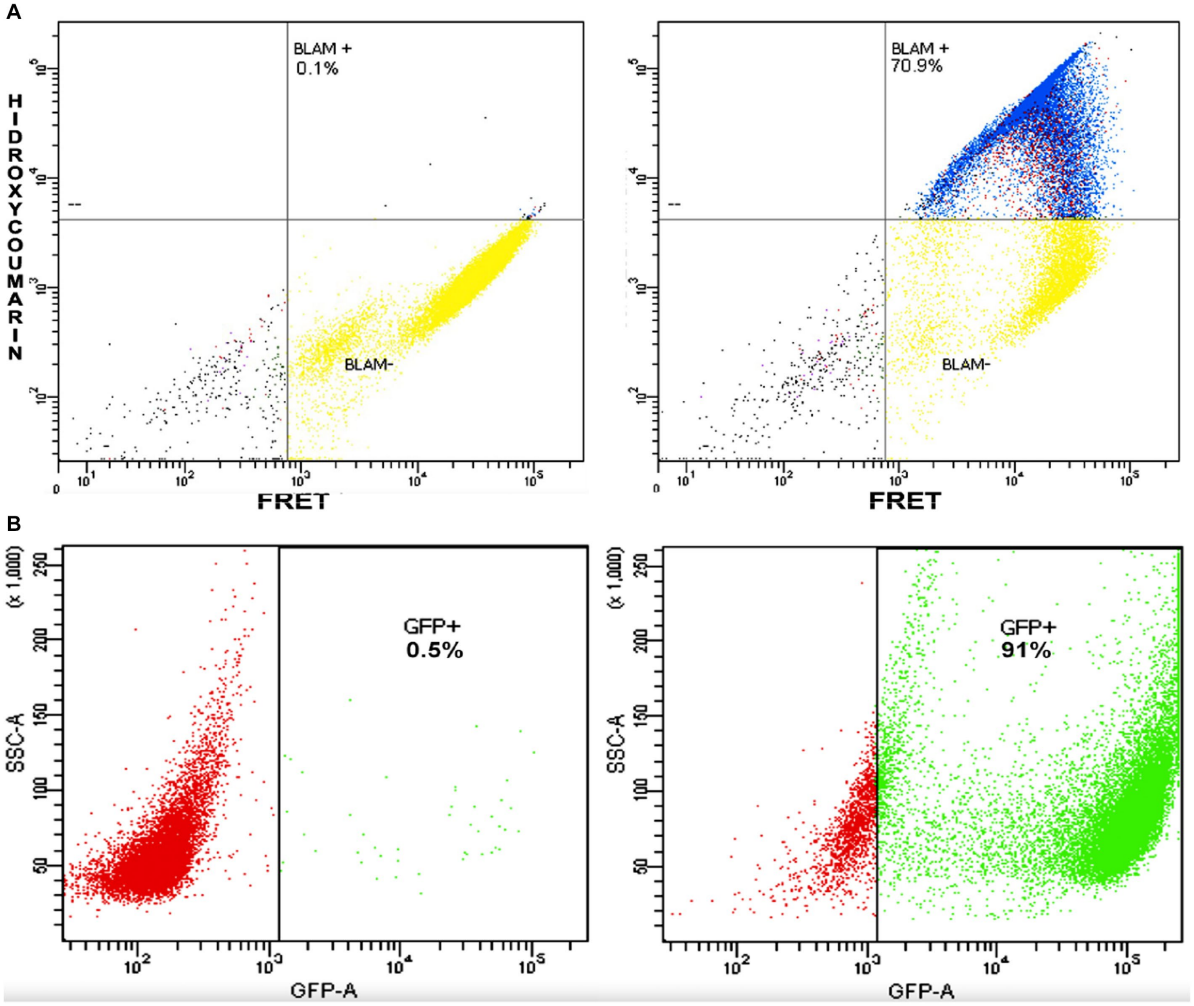
rgRSV virions carry the P-BlaM protein as a structural protein. **(A,B)** Flow cytometry analysis of beta-lactamase activity and reporter GFP expression. In **(A)**, the percentage of NHBE cells that shifted to the hydroxycoumarin emission channel as a result of infection at an equivalent dose of 1 virion per cell (1 MOI) for 2 h with rgRSV virions carrying P-BlaM in trans is shown. In **(B)**, the percentage of NHBE cells expressing GFP from an equivalent volume aliquot used in **(A)** is shown.

Given that the western blot suggested the presence of the P-BlaM as a structural protein in the virion, we proceeded to evaluate the ability of rgRSV virions to carry the P-BlaM protein, which had been stably expressed in HEp-2 cells transduced with the pLVX-P-BlaM lentivirus. The virions were then used to assess both entry (measured by hydroxycoumarin channel emission due to beta-lactamase activity) and infection (GFP expression) in HEp-2 cell cultures using flow cytometry (Figure 4A vs. Figure 4B). Remarkably, when using the same volume per infectious aliquot (at an equivalent dose of 1 virion per cell), 70.9% of the cells exhibited fluorescence in the hydroxycoumarin channel, providing evidence that the virions indeed carried the P-BlaM protein (Figure 4A). The viral aliquot titer, estimated through beta-lactamase activity, was determined to be 6.30 × 10^5^ FFU, while the titer estimated through GFP expression was 1.01 × 10^6^ FFU. Based on the above results, we confirmed the successful incorporation of the P-BlaM protein into the recombinant rgRSV-P-BlaM virion.

### Quercetin interferes with RSV entry

**Figure 5:**
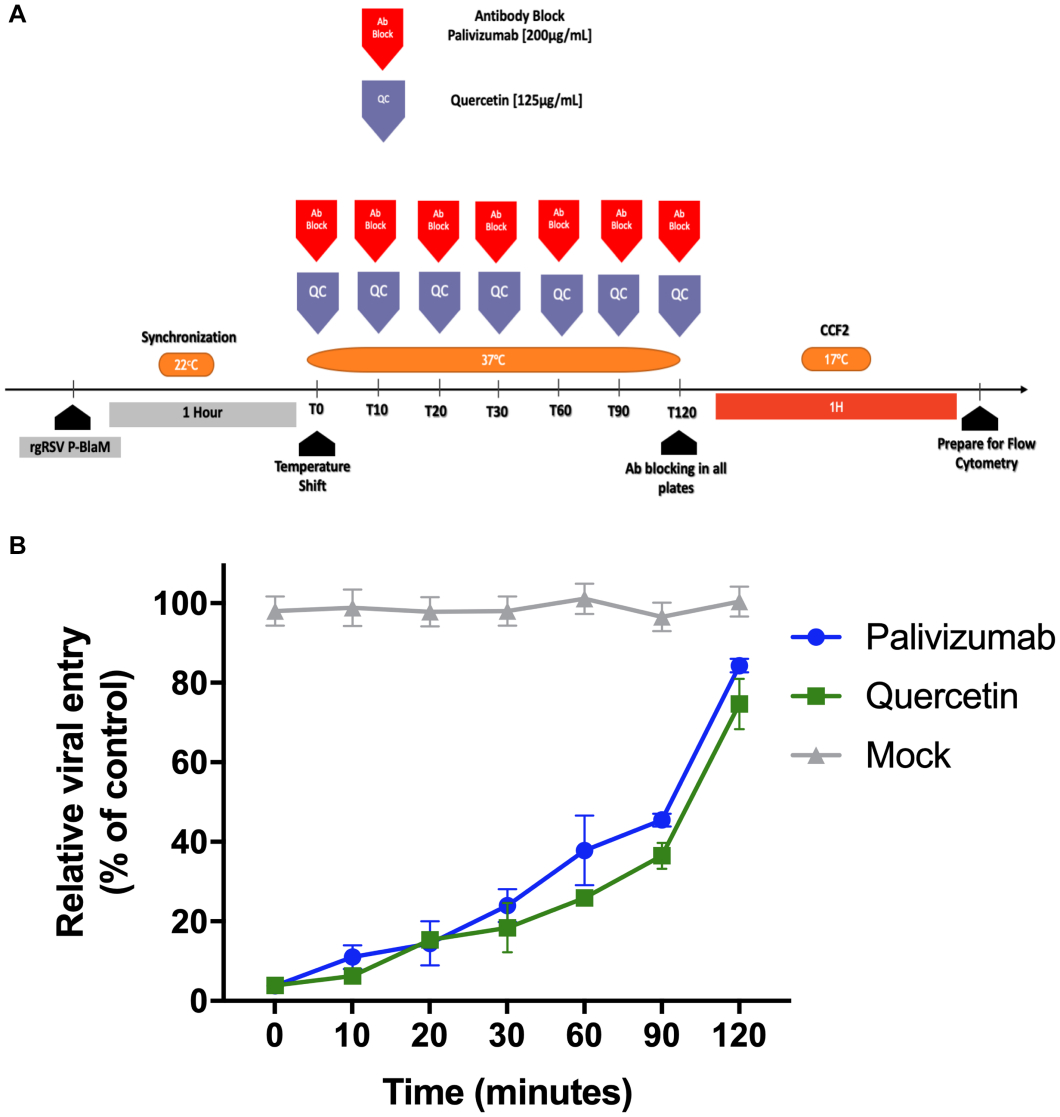
Time-of-addition assay of quercetin showed that it inhibits virion envelope fusion with the plasma membrane. **(A)** Experimental strategy diagram. After adsorbing the virions at 22°C to synchronize virion entry using at an infectious dose of 3 FFU per 10 cells, which is approximately 0.3 MOI, the cultures were shifted to 37°C. Sets of 3 cultures were treated with palivizumab or quercetin at 0, 10, 20, 30, 60, 90, and 120 min intervals. After 120 min, all cultures were loaded with CCF2-AM at 17°C for 1 h. Cultures were processed for flow cytometry, and the emission shift toward the hydroxycoumarin channel was determined. **(B)** Percentage of cells showing emission shift to the hydroxycoumarin channel. The presented results correspond to the mean ± SEM of three independent experiments.

Quercetin has been evaluated as an antiviral, particularly against RSV (Machado et al., 2019; Lopes et al., 2020). There are suggestions indicating that it plays a role in various stages of RSV infection, from adsorption to the membrane, to transcription, and viral replication. The role of quercetin during the virus envelope fusion event with the cell membranes of undifferentiated bronchial epithelium cultures was evaluated using a “Time-of-addition” (ToA) assay. The rgRSV-P-BlaM virions were adsorbed at 22°C for 1 h to synchronize the viral entry. The ToA assay started when the cultures were shifted to 37°C. From that moment, and every time point (0, 10, 20, 30, 60, 90, 120 min) for a period of 2 h (Figure 5A), different triplicate sets of cultures were exposed to quercetin or the palivizumab antibody. At the end of the 2 h, the cultures were incubated with CCF2-AM loading buffer for 1 h at 17°C, followed by the detection of the percentage of cells that emitted fluorescence in the hydroxycoumarin channel using flow cytometry. Quercetin inhibited viral entry, and the kinetics that followed were like those observed when the cultures were incubated with palivizumab (Figure 5B). The impact of quercetin on cellular viability was thoroughly evaluated in our study. Despite comprehensive analyses, the results consistently demonstrated no significant effects on cell viability (data not shown).

### Inhibition of ERK-1/2 activity interferes with RSV entry

**Figure 6:**
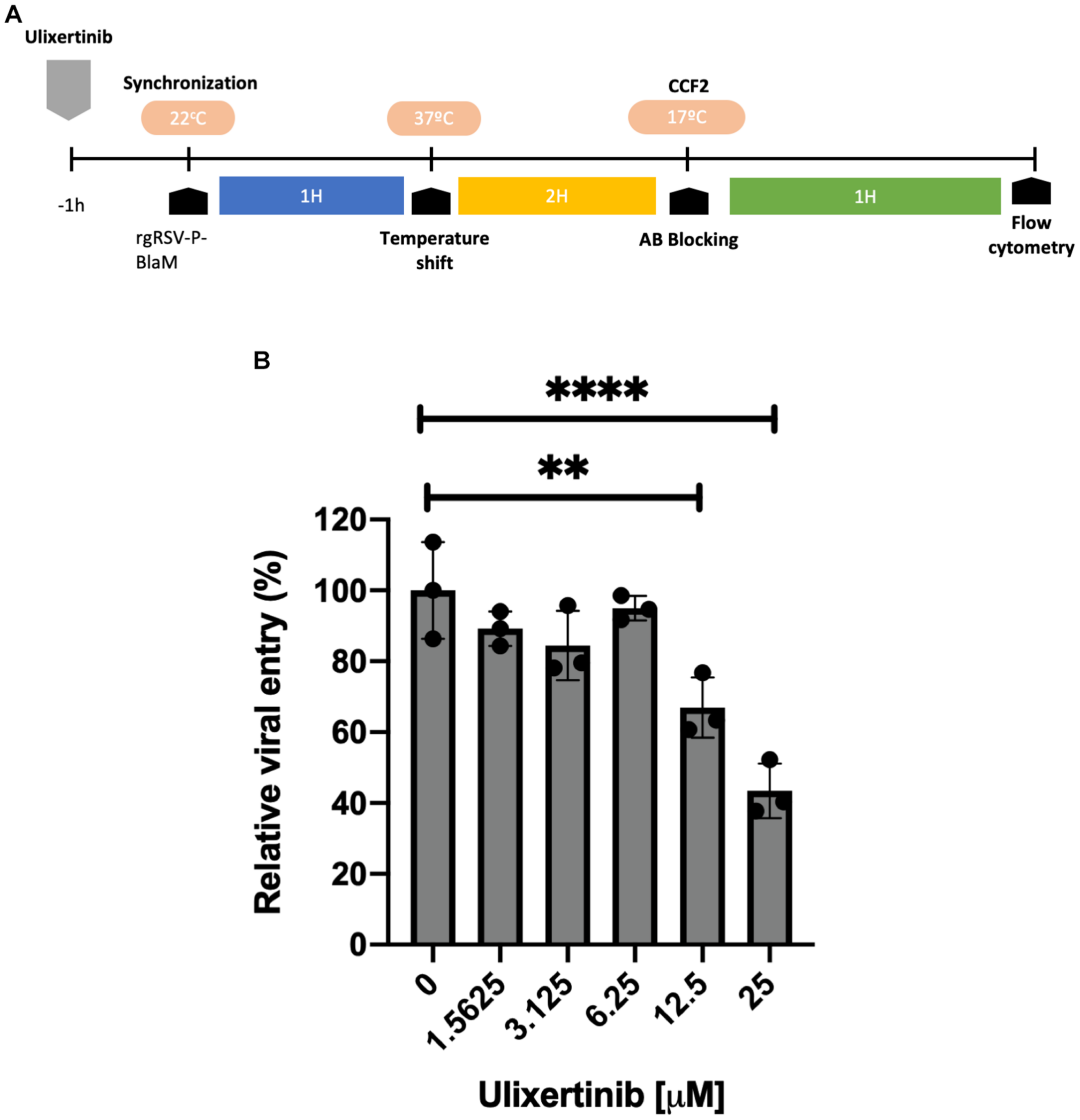
Dose-response evaluation of the ulixertinib inhibitor in viral entry. **(A)** Experimental strategy diagram. NHBE cell cultures were treated with different concentrations of ulixertinib. During inoculation with rgRSV-P-BlaM (at an infectious dose equivalent to 1 virion per 2 cells), the drug was present in the medium, and drug presence was maintained in the culture medium. The control consisted of cultures exposed to DMSO. **(B)** Percentage of cells showing emission shift to the hydroxycoumarin channel. At a concentration of 25 μM, there was a reduction of approximately 60% of the percentage of cells that shifted toward the hydroxycoumarin channel under control conditions (^****^p < 0.0001). The presented results correspond to the mean ± SEM of three independent experiments.

Previous studies have proposed that the signaling pathway leading to the activation of ERK-1/2 interferes with RSV infection, but it is not known whether active ERK-1/2 is required as part of the process or mechanism that facilitates RSV entry. Ulixertinib is a competitive ATP inhibitor at the active site of ERK-1/2, interfering with the phosphorylation of its substrates. In a dose-response assay, undifferentiated epithelial cell cultures were incubated with ulixertinib for 1 h before viral inoculation, and ulixertinib remained present throughout the incubation with the virus. The rgRSV-P-BlaM virions were adsorbed to the cell membrane for 1 h at 22°C to synchronize viral entry, and then cells were incubated at 37°C for 2 h. CCF2-AM loading and detection of hydroxycoumarin signal were performed as previously described. Ulixertinib at concentrations of 12.5 μM and 25 μM reduced the number of cells emitting hydroxycoumarin signal by up to 65 and 50%, respectively, compared to the cultures treated with DMSO (Figure 6). Consequently, the ERK-1/2 pathway seems to play an important role in facilitating RSV entry in a subset of cells. The impact of ulixertinib on cellular viability was thoroughly evaluated in our study. Despite comprehensive analyses, the results consistently demonstrated no significant effects on cell viability (data not shown).

## Discussion

In this report, we present the development of a recombinant virion that allows us to evaluate and determine the processes contributing to RSV entry. The rgRSV-P-BlaM recombinant virion carries the P-BlaM as a structural protein, enabling the detection of viral entry.

In the literature, various approaches have been described to evaluate viral entry of enveloped viruses, differing in speed (San-Juan-Vergara et al., 2012), sensitivity (Alonas et al., 2014), and precision (Kolokoltsov et al., 2007; Krzyzaniak et al., 2013). Some methods, such as the increase in fluorescence of secondary membrane fluorochromes due to FRET disruption (e.g., R18 and/or DiD), provide an overall quantitative assessment but are not efficient in discriminating based on cellular event (San-Juan-Vergara et al., 2012). Moreover, these fluorochromes can flow between adjacent membranes, leading to increased fluorescence and false-positive results during evaluation. Dual or discriminative immunofluorescence allows determination of the location where the fluorescence signal is occurring, whether in the cytoplasm, endosome, or another organelle (Yang et al., 2022). However, this approach uses statistical methods to quantify a region observed through the microscope.

Real-time tracking of each virion, using differential labeling to detect both the virion envelope and its content, allows the evaluation of movement speed and direction to infer whether the fusion of the virion envelope with cell membranes occurs at the plasma membrane or after endocytosis (San-Juan-Vergara et al., 2012; Alonas et al., 2014). However, quantification is limited to a small number of cells, and the association of speed and direction with endocytosis may be affected by subcortical membrane structures, potentially leading to misconceptions about the fusion site (de Armas-Rillo et al., 2016; Ripa et al., 2021).

Reporters expressing fluorescent or luminescent proteins under the control of viral polymerase have a delay of several hours (>12 h) before providing detectable signals, integrating processes such as adsorption, entry, viral content release, transcription and protein synthesis (Soboleski et al., 2005; Hotard et al., 2012; Wang et al., 2020). This delay makes it challenging to determine which cellular pathway is involved or which stage is affected by a drug (Falzarano et al., 2015).

Cavrois et al. (2002) developed a technique to evaluate viral entry based on beta-lactamase activity, released by a virion carrying the Vpr-BlaM chimeric protein. This allowed for the quantitative study of processes associated with HIV-1 entry. Pseudotyped virions carrying Vpr-BlaM have contributed to the understanding of fusion protein-mediated viral content release. However, the disadvantage of pseudotyped virions is that conclusions may be affected by the number of fusion proteins and accessory molecules present in the membrane or the lipid composition of the region where the particle assembles. We used different experimental approaches to develop and read the CCF2 cleavage-associated signal. When we used probenecid in primary cell cultures of NHBE cells, we noticed that the cells showed morphological changes and a significant fraction of them were detached, clearly indicating drug-induced toxicity. After reviewing the transcriptomic data published by Travaglini et al. (2020), we confirmed that NHBE cells did not express classical multidrug resistance genes such as MDR-1. Miyauchi et al. (2009) investigated the entry of HIV carrying Vpr-BlaM in different cell cultures and their experimental conditions avoided the use of probenecid and kept the CCF2-AM in the cell culture, modifying the original approach of Cavrois et al. (2002). Miyauchi et al. (2009) kept CCF2 at 13.5°C overnight to detect BlaM activity. In addition, lowering the temperature is also a mechanism that significantly slows down drug efflux, according to the manufacturer’s guidelines. Taking all of this into account, our final experimental conditions assessed BlaM activity in NHBE cell cultures at 17°C while maintaining CCF2-AM in the medium. It is important to note that in our experiments, virions were removed from the cell cultures before CCF2-AM loading medium was added to the cell cultures. In addition, according to the manufacturer’s instructions, CCF2 must be free of the AM moiety to become a substrate for BlaM activity; the AM moiety is removed by cytoplasmic esterases. Finally, we measure the shift to blue signal by flow cytometry, which requires that cells be detached, washed, and processed before they are ready for cytometry. Thus, our experimental conditions assess BlaM activity inside cells. Therefore, the virion of interest for studying the entry mechanism should preserve its native characteristics by carrying BlaM.

Based on available information, this is the first pneumovirus that has been successfully engineered to carry the BlaM protein by fusing with RSV-P phosphoprotein. Additionally, modeling of the P-BlaM chimera showed that it retains the same regions of secondary structure modeled from the phosphoprotein sequence. Coupled with the fact that RSV-P is an intrinsically disordered protein capable of adapting to interact with other proteins, this provides opportunities for its interaction with other RSV-P protein monomers, allowing it to be loaded as a structural protein within the virion. Furthermore, this ensures that the bound BlaM protein does not interfere with the proper interaction of RSV-P with the polymerase. This approach can be used to study the entry mechanism of metapneumoviruses. Similarly, the structure of phosphoproteins from virus species belonging to the paramyxovirus genus suggests that they are intrinsically disordered proteins. The RSV-P protein is an essential factor in the assembly of the RNA-dependent transcription complex (Grosfeld et al., 1995; Yu et al., 1995; García-Barreno et al., 1996). In the absence of RSV P protein, there is no viral transcription. Therefore, the GFP reporter expression shown in Figures 2, 4 and the fact that we have grown recombinant RSV-P-BlaM clearly support the presence of the P protein within the virions. It is expected that not all virions will simultaneously carry both RSV P protein and P-BlaM as shown in Figures 2, 4. However, the fraction of virions carrying P-BlaM or both proteins is high enough to perform the assays and draw the appropriate conclusions. Although it would be a very good idea to assess the fraction of virions carrying both P and P-BlaM, the use of P-specific antibodies would not allow us to answer whether there is functional competition between P and P-BlaM. Unfortunately, Western blot is a biochemical technique that requires the contents of all virions to be mixed before the assay is performed.

Several reports in the literature support that the ToA approach can be used to determine the step at which the drug or compound inhibits viral infection. Daelemans et al. (2011) demonstrated the antiviral mechanism of action of several drugs against HIV infection. In addition, Daelemans et al. (2011) and Aoki-Utsubo et al. (2018) showed that all drugs with the same mechanism of inhibition clustered in the same time window. In the case of RSV, Mackman et al. (2015) reported that the fusion inhibitor GS-5806 was only effective when added with the viral inoculum. In addition to GS-5806, Perron et al. (2016) reported similar results for the compound VP-14637, a known fusion inhibitor. Wang et al. (2021) reported that a triazole oxadiazole derivative showed antiviral activity even when administered 8 h after viral entry, which was associated with a post-entry mechanism of action targeting viral transcription. Fu et al. (2019) reported that azathioprine and 6-mercaptopurine blocked RSV when added up to 6 and 8 h after infection, consistent with a post-entry mechanism. These 2 compounds interfered with RSV transcription. In addition, they tested cyclopiazonic acid and found that this drug also showed antiviral effect when added up to 8 h after infection, compatible with its already known mechanism targeting viral transcription. On the other hand, when they tested a fusion inhibitor such as GS-5806, it rapidly lost its antiviral effect even when added 2 h after infection. Sudo et al. (2005) reported that the small molecule YM-53403 was effective when added up to 8 h after RSV infection, which was associated with targeting viral transcription. In addition, Sudo et al. (2001) also reported that benzodithiin interfered with RSV replication even when administered 16 h after infection. Sudo found that this compound interfered with the intracellular processing of the RSV fusion protein, resulting in the production of inactive viruses.

The use of rgRSV-P-BlaM virions using the ToA approach confirmed that quercetin interferes with the mechanism of viral content release. Flavonoids, such as quercetin, possess many properties, including anti-inflammatory, estrogenic, antimicrobial, antiallergic, antioxidant, vascular activity, and antitumor/cytotoxic effects (Elliott Middleton et al., 2000; Harborne and Williams, 2000). Lopes et al. (2020) reported that quercetin inhibited the adhesion step during the viral replication cycle, but our findings differ, clearly, we showed that viral entry kinetics inhibition of quercetin overlapped with the inhibition curve observed when using palivizumab. This is relevant because our experimental design ensured that virions were adsorbed to the cell membrane before quercetin administration and that palivizumab interferes with the fusion mechanism dependent on the RSV F protein.

Furthermore, we showed that the ERK-1/2-dependent pathway may play a facilitating role in the entry in undifferentiated bronchial epithelial cells. ERK-1/2 activation and its dependence are involved in various contexts during infection, including the innate immune response through the production of chemokines and cytokines, which are necessary for infection and viral replication (Chen et al., 2000; Pinto et al., 2011). However, it has not been demonstrated whether ERK-1/2 inhibition could affect virion entry. Palivizumab, which we used in time-of-addition (ToA) assays, demonstrated that BlaM delivery to cells required fusion of the viral envelope with the cell membrane and was therefore not the result of a cell-mediated process triggered by the switch to 37°C (Figure 5). Palivizumab is a humanized monoclonal antibody that targets the RSV F protein and prevents viral fusion. Palivizumab, which was added at the time the cultures were shifted to 37°C, prevented RSV entry. Ulixertinib is a highly specific inhibitor that targets the active site of ERK-1/2. Although off-target effects may occur, rgRSV-P-BlaM serves as a useful tool to explore the involvement of kinases during RSV entry.

Consequently, this virion provides an excellent tool for studying the cellular processes involved in RSV entry and fusion mechanisms, drug screening and discovery to interfere with entry and fusion, as well as the development of vaccines, whether directly using attenuated virions, evaluating escape mutations, or using it as a mechanism for modified microneutralization assays.

## Data availability statement

The raw data supporting the conclusions of this article will be made available by the authors, without undue reservation.

## Ethics statement

Ethical approval was not required for the studies on humans in accordance with the local legislation and institutional requirements because only commercially available established cell lines were used. Ethical approval was not required for the studies on animals in accordance with the local legislation and institutional requirements because only commercially available established cell lines were used.

## Author contributions

MÁ-A: Conceptualization, Data curation, Formal analysis, Investigation, Methodology, Resources, Validation, Visualization, Writing – original draft, Writing – review & editing. JV-C: Formal analysis, Investigation, Methodology, Resources, Validation, Visualization, Writing – original draft, Writing – review & editing. CC-C: Investigation, Methodology, Validation, Visualization, Writing – original draft, Writing – review & editing. LH-G: Writing – review & editing. HC: Methodology, Writing - review & editing. AR: Writing – original draft, Writing – review & editing. FB-F: Writing – original draft, Writing – review & editing. AB: Investigation, Writing – original draft, Writing – review & editing. MK: Investigation, Methodology, Writing – review & editing. AM: Funding acquisition, Investigation, Writing – review & editing. MP: Conceptualization, Formal analysis, Funding acquisition, Investigation, Methodology, Resources, Supervision, Writing – original draft, Writing – review & editing. HS-J-V: Conceptualization, Data curation, Formal analysis, Funding acquisition, Investigation, Methodology, Project administration, Resources, Supervision, Validation, Visualization, Writing – original draft, Writing – review & editing.
